# Supplementary material for: Maternal Diet High in Linoleic Acid Alters Renal Branching Morphogenesis and mTOR/AKT Signalling Genes in Rat Fetal Kidneys
Source: Int J Mol Sci. 2024 Apr 25;25(9):4688. doi: 10.3390/ijms25094688 (PMC11083378; doi:10.3390/ijms25094688)
Supplement: Supplementary file 1 [file ijms-25-04688-s001.zip › ijms-2962070-supplementary.pdf]

Supplementary file

Table S1: Accession numbers and targets analysed by real time PCR

mRNA	RefSeqID
Ret	NM_001110099
Gdnf	NM_019139
Tgfb1	NM_021578
Gfra1	NM_012959
Lepr1	NM_012596
Jak2	NM_031514
Stat3	NM_012747
Stat5a	NM_017064
Lrp2	NM_030827
mTOR	NM_019906
Pi3kca	NM_133399
Prkaa1	NM_019142
Prkab2	NM_022627
Akt3	NM_031575
Flt1	NM_019306
Vegf	NM_001110334
